# Inflammation in Hypervolemic Hemodialysis Patients: The Roles of RelB and Caspase-4

**DOI:** 10.3390/ijms242417550

**Published:** 2023-12-16

**Authors:** Christof Ulrich, Zeynep Canim, Eva Herberger, Matthias Girndt, Roman Fiedler

**Affiliations:** Department of Internal Medicine II, Martin Luther University Halle-Wittenberg, 06120 Halle (Saale), Germany; zeynep611@live.de (Z.C.); evaherberger@gmx.de (E.H.); matthias.girndt@uk-halle.de (M.G.); roman.fiedler@uk-halle.de (R.F.)

**Keywords:** hypervolemia, HD, caspase-4, RelA, RelB, endotoxin, inflammation

## Abstract

Hypervolemia is associated with inflammation in hemodialysis (HD) patients. How hypervolemia triggers inflammation is not entirely known. We initiated a cross-sectional study enrolling 40 hemodialysis patients who were categorized into normovolemic (N; 23) and hypervolemic (H; 17) groups by bioimpedance measurement. A caspase activity assay in combination with a specific caspase-4 inhibitor was used to detect caspase-4 activity in isolated peripheral blood mononuclear cells (PBMCs). Transcription factors RelA (pS529) and RelB (pS552) were analyzed by phospho-flow cytometry. Serum endotoxins were detected by an amebocyte lysate-based assay, and *IL-6* (interleukin-6) and *TNF-α* (Tumor necrosis factor-α) gene expression were detected using the ELISA technique. Hypervolemic patients were older, more frequently had diabetes and showed increased CRP and IL-6 levels. Caspase-4 activity, which is linked to intracellular endotoxin detection, was significantly elevated in H patients. While the frequency of RelA-expressing immune cells and the expression density in these cells did not differ, the monocytic frequency of cells positively stained for RelB (pS552) was significantly decreased in H patients. Increased caspase-4 activity in H patients may indicate a cause of inflammation in H patients. The post-translational modification of RelB (pS552) is linked to downregulation of NF-kB activity and may indicate the resolution of inflammation, which is more distinct in N patients compared to H patients. Therefore, both higher inflammatory loads and lower inflammatory resolution capacities are characteristics of H patients.

## 1. Introduction

Hypervolemia is very common in patients with advanced chronic kidney disease (CKD), particularly in HD patients, 40 to 50% of whom were found to be overhydrated [1,2]. However, not only patients on maintenance dialysis are affected by volume overload. During earlier stages of kidney failure (stages 4 and 5), the incidence of overhydration is high [3]. Hyperhydration increases morbidity and symptom load in these patients [4]. Kidney and heart disease are often related to each other, and chronic heart failure is often accompanied by kidney failure [5]. In addition to overhydration and cardiovascular complications, inflammation is another piece in the uremic puzzle. It is widely accepted that inflammation plays a pivotal role in the progression of atherosclerotic disease in CKD [6]. Overhydration due to heart and kidney failure may foster systemic inflammation. Although the mechanisms have not been fully elucidated, “the intestinal mucosal permeability theory (IMP theory)” provides an attractive explanation [7]. It suggests that hypervolemia and mucosal swelling contribute to the impairment of barrier structure; alter the intestinal microbial flora; and induce inflammation by enabling the influx of endotoxin and microbiome-derived toxins such as indoxyl sulfate to the circulation [7,8,9], which may trigger inflammation. Markers of inflammation such as C-reactive protein (CRP) and interleukin-6 (IL-6) were found to be mostly increased in overhydrated CKD patients, while the data on tumor necrosis factor-alpha (TNF-α) were inconsistent [2,10]. Following the IMP theory, penetration of endotoxin from the intestine into the blood might be one of the main stimuli triggering TNF-α production [11]. Hung et al. demonstrated that TNF-α was elevated in CKD patients with volume overload [2]. The authors further speculated that CKD patients share some similarities with heart failure patients regarding fluid disbalance and cardiovascular mortality. Due to animal studies in which volume overload was associated with elevated expression of TNF-α in the heart [12], it appears possible that TNF-α is an important pathophysiological trigger under hypervolemic conditions. Thus, there are some arguments as to why hypervolemia could be associated with elevated TNF-α-mediated effects in HD.

Since serum TNF-a levels may vary, it may be helpful to look at its downstream effects. In this context, nuclear factor-kappa B (NF-kB) signaling is of eminent importance because transcription factors of this family play essential roles regarding innate and adaptive immunity. The mammalian NF-kB family comprises five members: RelA (p65), RelB, c-Rel, p105/50 and p100/52. It is known that TNF-α is able to activate the so-called classical NF-kB pathway [13]. In doing so, TNF-α phosphorylates RelA on serine 529, thereby increasing its transcriptional activity [14,15]. Other members of the TNF-α family, i.e., CD40, B-cell-activating factor receptor (BAFFFR), lymphotoxin ß receptor (LTßR) and receptor activator of NF-kB (RANK), can induce the so-called alternative NF-kB pathway by processing of p100 and liberation of RelB. After stimulation, RelB is very quickly phosphorylated at serine 552, which is the starting point for proteasomal degradation of NF-kB. The classical NF-kB pathway is believed to be involved in innate immunity, whereas the alternative pathway seems to be linked to B cells, dendritic cell maturation and lymphoid organogenesis [16]. Even more importantly, it seems to offer a way to control NF-kB activity [17]. Regarding the inflammatory nature of hypervolemia, it seems most likely that both NF-kB pathways are significantly influenced by fluid overload.

With regard to hypervolemic inflammation, it also appears to make sense to study caspase-4 activity. In the context of hypervolemia, we previously reported another inflammatory caspase, namely caspase-1 [18]. We clearly concluded that this caspase was not activated in overhydrated end-stage renal failure patients. Caspase-4, however, is a sensor of intracellular lipopolysaccharide (LPS). As LPS is too large to cross cell membranes by itself, outer-membrane vesicles, which are products naturally secreted by Gram-negative bacteria, are believed to function as cytosolic LPS delivery vehicles [19]. Analysis of caspase-4 activity could be a promising tool to illuminate the origin of hypervolemic inflammation. Nonetheless, small amounts of endotoxin are difficult to detect. Thus, this study can provide further insight into the inflammatory nature of chronic kidney disease evoked by hypervolemia.

## 2. Results

In this study, we stratified 40 chronic hemodialysis patients according to their fluid status as determined by bioelectric impedance vector analysis (BIVA) [20,21]. This method is an effective tool to examine the hydration status of HD patients. Defined by two parameters, i.e., resistance (ohm/m) and reactance (ohm/m), BIVA provides clues not only with respect to tissue hydration but also cell mass.

### 2.1. Overhydration Is Assoiated with Older Age, Diabetes and Inflammation

Lower reactance (N: 55 ± 13 vs. H: 30 ± 8, *p* < 0.001) and lower resistance values (N: 616 ± 117 vs. H: 471 ± 74, *p* < 0.001) are characteristics of hypervolemic HD patients. Overhydrated patients were significantly older, were more frequently diabetics and had a higher inflammatory burden. Furthermore, hypervolemic patients had a slightly higher BMI and systolic blood pressure values (Table 1). Regarding the electrolyte status, there were no differences between N and H patients (bicarbonate (mmol/L): 23.8 ± 2.0 vs. 22.6 ± 2.6, *p* = 0.135; sodium (mmol/L): 136.3 ± 2.5 vs. 136.6 ± 2.9, *p* = 0.969; calcium (mmol/L): 2.2 ± 0.2 vs. 2.2 ± 0.2, *p* = 0.792; potassium (mmol/L): 5.6 ± 0.8 vs. 5.8 ± 0.8, *p* = 0.459).

### 2.2. Granulocyte Numbers Are Elevated in Hypervolemia

On the cellular level, the inflammatory nature of hypervolemia is reflected by increased numbers of CD45-positive leucocytes. Among the different leucocyte subsets, only granulocyte numbers trended to be higher in overhydrated patients (Table 2).

### 2.3. IL-6 but Not TNF-α mRNA Expression Is Elevated in Hypervolemia

**Figure 1:**
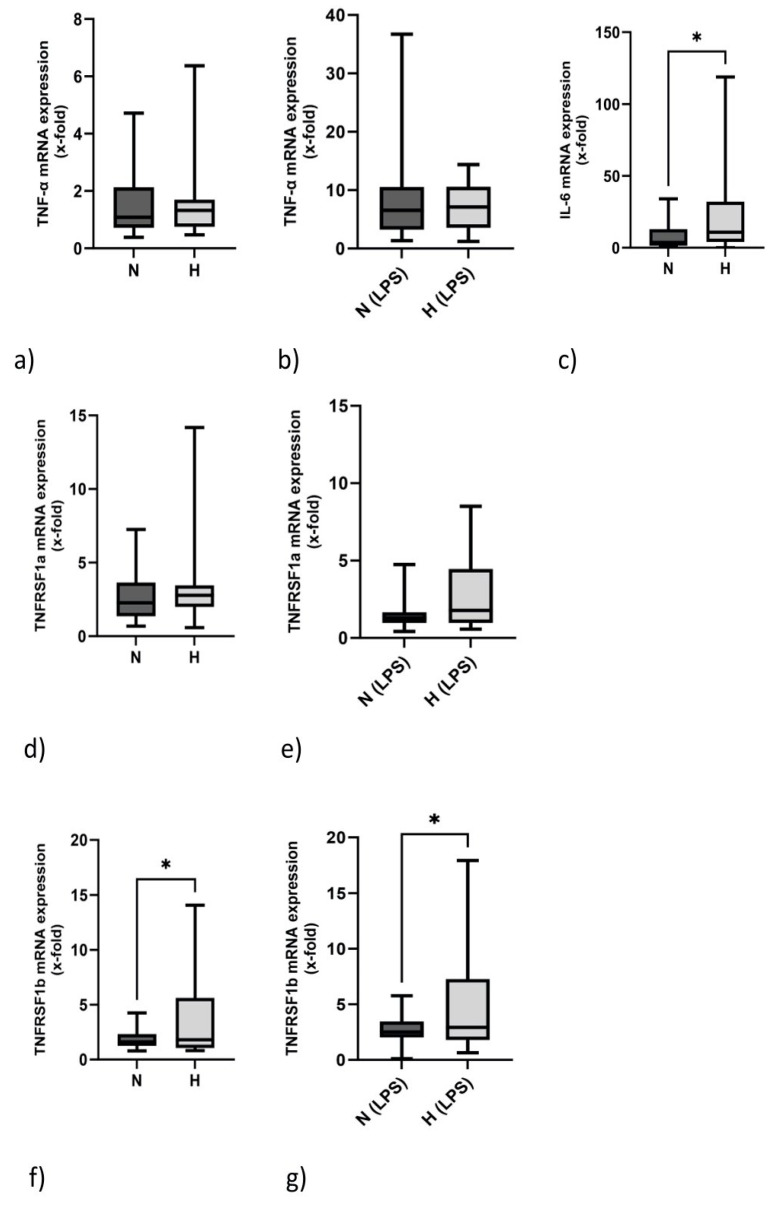
mRNA expression in PBMCs of normo- (N) and hypervolemic (H) HD patients: TNF-α mRNA expression under basal (**a**) and stimulated conditions (**b**). Comparison of IL-6 mRNA expression in unstimulated PBMCs (**c**). TNFRSF1a expression in unstimulated PBMCs (**d**) and upon LPS stimulation (**e**). TNFRSF1b mRNA expression under basal (**f**) and stimulated conditions (**g**). Data are presented as box plots with medians and 25/75 percentiles. Data were analyzed using Mann–Whitney tests. * *p* < 0.05.

Regarding the inflammatory nature exerted by hypervolemia in HD, we examined the mRNA expression of proinflammatory genes TNF-α and IL-6 in PBMCs. As demonstrated in Figure 1a, TNF-α mRNA expression did not differ between normovolemic and hypervolemic patients. The mRNA of this proinflammatory gene could be significantly induced by LPS in N and H patients (Figure 1b). In contrast to TNF-α, the mRNA expression of IL-6 was significantly upregulated in H patients (Figure 1c). Figure 1d,e demonstrate that TNFR1 mRNA expression is similar in N and H patents with and without LPS stimulation. TNFR1 transcripts are slightly downregulated by LPS (N: 2.7 ± 1.9 vs. 1.5 ± 1.1, *p* < 0.001; H: 3.2 ± 3.1 vs. 2.6 ± 2.5, *p* = 0.117). TNFR2 mRNA expression, however, is elevated in PBMCs of H patients. This holds true for unstimulated (Figure 1f) and LPS-stimulated samples (Figure 1g). Interestingly, mRNA expression of the p40 subunit of the proinflammatory IL-12 gene (IL-12B, IL-12p40), a gene which is primarily produced by monocytes and neutrophils and which is involved in NK-cell and CD4 helper-cell activation, also tended also to be higher in H compared to N patients (H: 6.9 (range: 1.1–186.5) vs. N: 3.4 (range: 0.6–27.0), *p* = 0.201).

### 2.4. IL-6 but Not TNF-α Protein Expression Is Elevated in Hypervolemia

**Figure 2:**
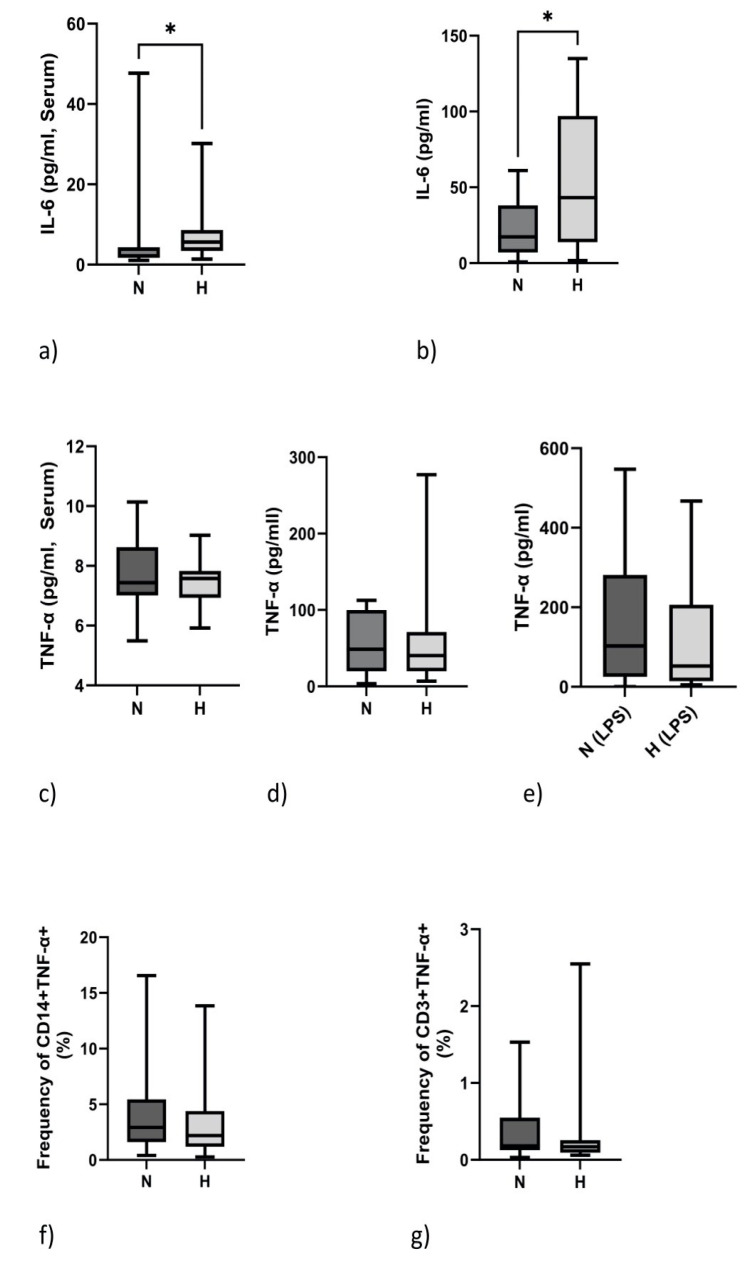
IL-6 and TNF-α protein expression in normovolemic (N) and hypervolemic (H) HD patients. IL-6 protein expression in serum (**a**) and supernatants of unstimulated PBMCs of N and H patients (**b**). TNF-α protein levels in serum (**c**) or PBMC supernatants of N and H patients (**d**). TNF-α expression upon LPS stimulation (**e**). Frequency of monocytes (**f**) and lymphocytes (**g**) positively stained for TNF-α. Data were analyzed using Mann–Whitney tests. * *p* < 0.05.

Corresponding to the mRNA expression data, IL-6 protein expression was increased in the serum and in supernatants of cultured PBMCs of H patients (Figure 2a,b). In contrast, TNF-α protein expression did not differ between N and H patients (Figure 2c,d). As expected, neither TNF serum nor levels in culture supernatants differed between N and H patients (Figure 2c,d). TNF-α protein content could be increased by stimulation with LPS (Figure 2e). As TNF-α can be produced by monocytes and lymphocytes, we analyzed the cells using flow cytometry. Indeed, the frequency of monocytes, as well as that of lymphocytes, positively stained for TNF-α did not differ between the two groups (Figure 2f,g).

### 2.5. Monocytic Transcription Factor RelB (pS552) but Not RelA (pS529) Differs between Normo- and Hypervolemic Patients

**Figure 3:**
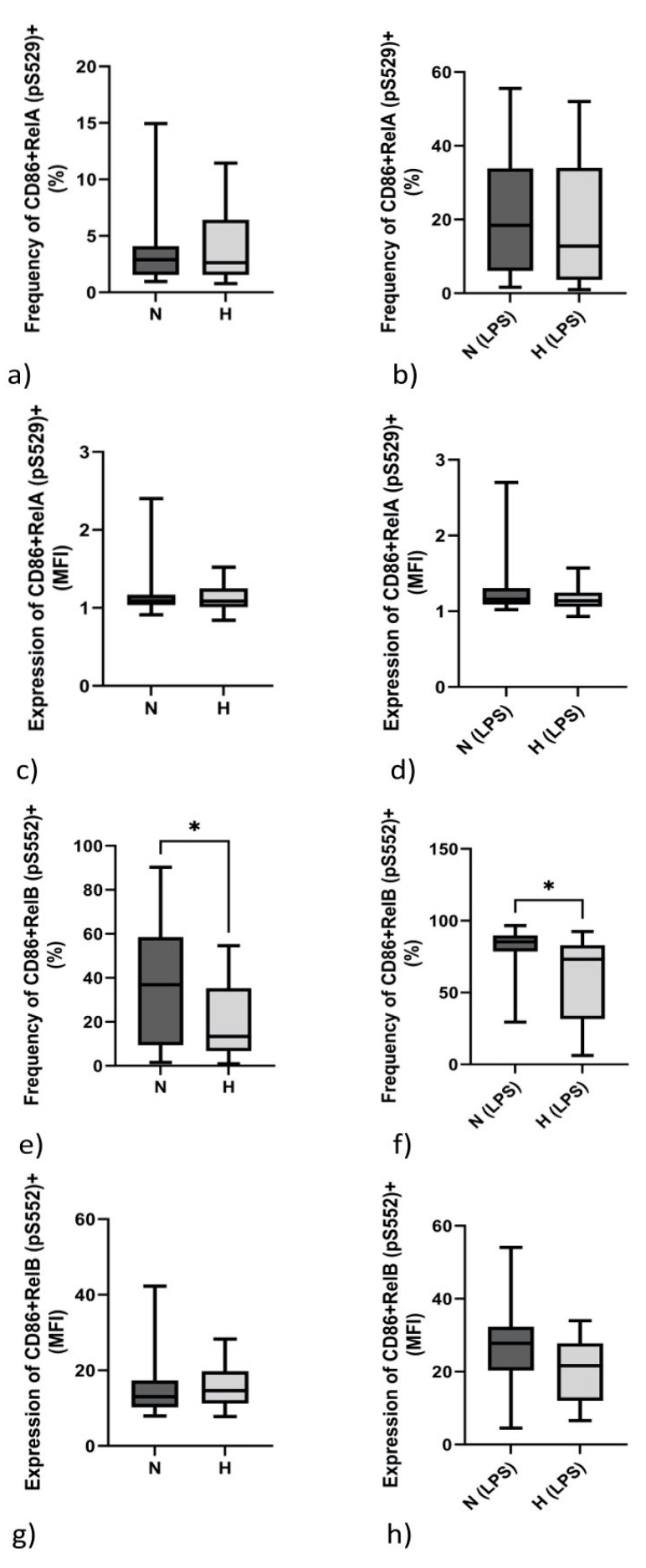
Expression of Rel B but not the phosphorylation of Rel A differs between N and H patients. The frequency of unstimulated monocytes (CD14+) positively stained for RelA phosphorylated at serine P-529 (**a**), as well as the median fluorescence intensity (MFI) of phosphorylated RelA (pS529), did not differ between the PBMCs of N and H patients (**c**). This was also true when PBMCs were stimulated with LPS (**b**,**d**). In contrast to RelA, the frequency of monocytes positively stained for RelB (pS552) was significantly enhanced under basal conditions (**e**) and upon LPS stimulation (**f**). RelB expression per cell (MFI) did not differ between N and H patients (**g**,**h**). Data were analyzed using Mann–Whitney tests. * *p* < 0.05.

The NF-kB family is a crucial player in innate and adaptive immunity. Its members, i.e., RelA and RelB, fulfill different tasks. Since TNF-α is usually able to activate RelA by phosphorylation, we analyzed the phosphorylation at serine residue 529 (pS529). As demonstrated in Figure 3, neither the frequency of CD86+RelA (pS529)+ monocytes nor the density of expression per cell (MFI) differed in unstimulated (Figure 3a,c) or LPS-stimulated (Figure 3b,d) PBMCs between N and H patients.

In contrast, the frequency of RelB (pS552)-positive monocytes was elevated in N patients both at the basal level (Figure 3e) and upon LPS stimulation (Figure 3f). Regarding the density of expression of RelB, no difference between N and H patients was observed (Figure 3g,h).

Transcription factor analysis in CD4+ lymphocytes revealed that RelA (pS529) did not different between the two cohorts under either unstimulated or stimulated conditions (Table 1). RelB (pS552) staining revealed a higher percentage of CD4+ReLB+ cells in N patients upon LPS stimulation of PBMCs (Table 3).

### 2.6. Caspase-4 Activity but Not the Serum Endotoxin Level Is Elevated in Hypervolemia

**Figure 4:**
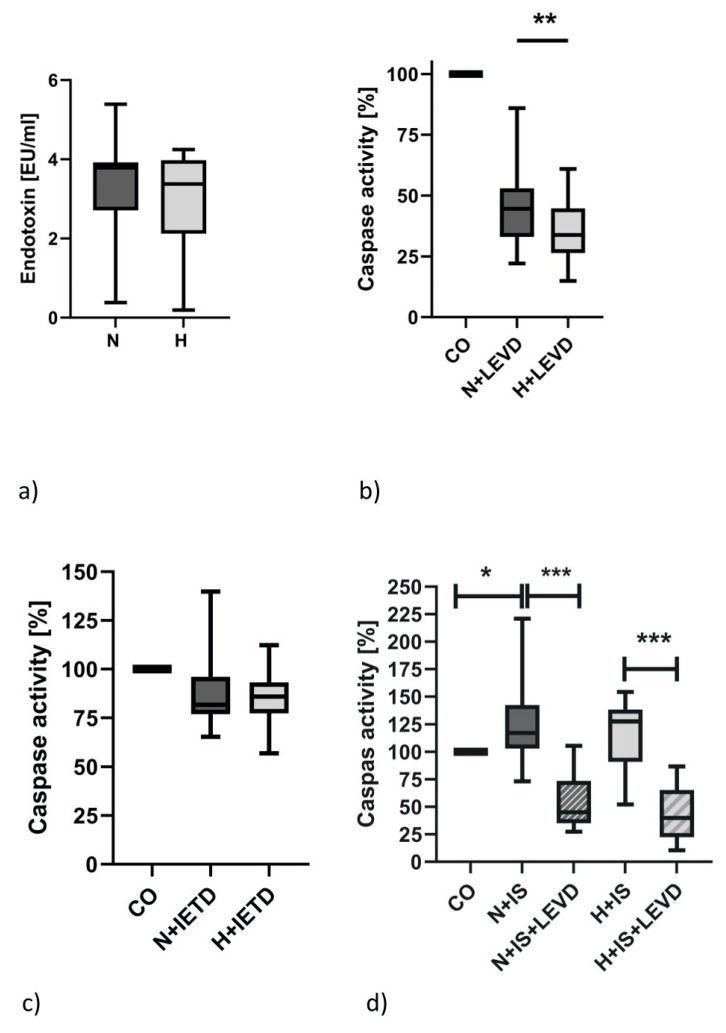
Endotoxin levels and caspase-4 activity in normovolemic (N) and hypervolemic (H) patients: Endotoxin level in serum of N and H patients (**a**). Caspase-4 activity in N (N+LEVD) and H (H+LEVD) patients (**b**). Caspase-8 activity in N (N+IETD) and H (H+IETD) patients (**c**). Induction of caspase activity by indoxyl sulfate (IS) and specific inhibition of this caspase activity with caspase-4 specific inhibitor Ac-LEVD-CHO in N and H patients (**d**). Data were analyzed using Mann–Whitney tests or one-way ANOVA. * *p* < 0.05; ** *p* < 0.01; *** *p* < 0.001.

Regarding the putative inducers of inflammation, we examined whether endotoxin—possibly as a consequence of a different “intestine leakage phenomenon”—could be found to be elevated in hypervolemic patients. As demonstrated in Figure 4a, the serum endotoxin levels did not differ between the two patient cohorts. Thus, we speculate that our endotoxin measurement is not sensitive enough to detect small differences between groups. It has been known for a long time that an inflammatory caspase, namely caspase-4, is able to detect small amounts of intracellular endotoxin. The caspase assay we used can detect activities of inflammatory caspases 1, 4 and 5, while the distinction between the specific actions of these caspases is made by the use of specific caspase inhibitors. As shown in Figure 4b, the caspase activity can be suppressed by 52 + 17% in N patients and by 65 + 17% in H patients using the caspase-4-specific inhibitor. This, of course, means that caspase-4 is more active in H patients. However, we have to be aware that the substrate specificities of the inhibitors are overlapping. This is demonstrated in Figure 4c. Using the caspase-8-specific inhibitor (Ac-IETD-CHO), in our caspase assay, we see a small inhibitory effect that is quite similar between the two groups.

Stimulating PBMCs with 1 mmolar indoxyl sulfate increased caspase-4 activity significantly in N patients and to a minor degree in H patients (Figure 4d). The relative specificity of this action was proven by the use of the caspase-4 inhibitor (Ac-LEVD-CHO). However, we also have to keep in mind that 1 mmolar indoxyl sulfate is the highest amount of the uremic toxin ever found in dialysis patients. Typically, the plasma levels are in the µmolar range.

## 3. Discussion

Patients suffering from CKD are at high risk for hypervolemia [22,23]. This coincides with an inflammatory state prevailing in these patients [24]. Thus, high levels of proinflammatory markers such as IL-6, CRP and TNF-α have been reported in overhydrated CKD patients [2,25]. Regarding TNF-α, this cross-sectional study provides no indications that hypervolemia may cause relevant differences in normo- and hypervolemic patients. TNF-α mRNA and protein levels, as well as the frequency of TNF-α-positive monocytes and lymphocytes as main TNF-α producers, do not differ between N and H patients. TNF-α induces signaling by binding to two different receptors—TNFRS1a (TNFR1) and TNFRS1b (TNFR2). While TNFR1 is believed to mediate inflammation and cell death, TNFR2 is mainly associated with homeostatic bioactivities. Furthermore, unlike TNFR1, which is located on the surface of most cells, TNFR2 is mainly expressed by immune cells. It is of note that TNFR2 is elevated in H patients. This receptor is associated with homeostatic activities, including tissue regeneration, cell proliferation and cell survival [26]. Our TNF-α study results are in the line with Beberashvilli and colleagues [10], who did not find a significant change in TNF-α in a study including 250 hypervolemic CKD5-D patients. Of course, we have to keep in mind that our study group was small. Conversely, Hung and coworkers, including 338 chronic kidney disease patients in stages 3–5 in their study, demonstrated that hypervolemic patients not only had elevated IL-6 levels but also significantly increased TNF-α levels [2]. Interestingly, we could not find this relationship in patients on maintenance dialysis (CKD5-D). Thus, further studies are necessary to examine if TNF-α is elevated in hypervolemic patients with chronic kidney disease.

mRNA data are supported by analysis of NF-kB transcription factor members RelA and RelB. TNF-a belongs to the group of cytokines that can induce NF-kB signaling [27,28]. TNF-α is able to phosphorylate RelA on serine at position 529 [15]. Phosphoprotein analysis of RelA revealed that neither the frequency nor the expression density of RelA (pS529) differs between N and H patients. Therefore, in contrast to IL-6, TNF-α levels appear not necessarily to be elevated in hypervolemic patients. Unlike RelA, RelB (pS552) is significantly elevated in the monocytes of normovolemic patients. Given a study conducted by Marienfeld et al., it is clear that RelB is not only linked to dendritic cells (DCs) but also to B-cell maturation. Post-translational modification of RelB—phosphorylation on serine at position 552 (pS552)—appears to be a signal for the degradation of RelB, limiting NF-kB activity [23,29]. Furthermore, RelB-containing dimers have DNA-binding specificity, and it is known that RelB recruitment to the proinflammatory *IL-12B* (IL-12-p40) gene is associated with transcriptional downregulation [30]. In line with this argument, we also measured lower IL-12B mRNA levels in N patients, which favors the hypothesis that RelB has an anti-inflammatory capacity and that regulation of NF-kB-induced gene expression is dependent on the balance of different NF-kB species [23,31].

To glean insights on the putative origins of hypervolemic inflammation, we measured the levels of endotoxin and caspase-4 activity in both patient groups. We failed to detect differences in serum endotoxin levels between the two groups. However, it has long been known that hemodialysis initiation is associated with elevated endotoxin levels [8]. Caspase-4 activity is enhanced by intracellular LPS. The elevated caspase-4 activities found in H patients may be an indication of a higher intracellular bacterial load in these patients. It is not clear at the moment why elevated caspase-4 activity levels are not simultaneously linked to higher serum endotoxin levels. At some stage, cells “infected” with endotoxin activate pyroptotic programs and are doomed to death. As a consequence, endotoxin would be released. This discrepancy must be investigated in future studies. Furthermore, we have to keep in mind that caspase-4 activity can also be induced by other molecules. In a recent paper, we demonstrated that caspase-4 activity can be induced by a high concentration (1mmolar) of uremic toxin indoxyl sulfate in the monocytic THP-1 cell line [32]. It seems plausible that if these results can be stably transferred from THP-1 cells to PBMCs of CKD patients, caspase-4 activity can be stimulated by quite different substances. In this study, we confirmed that the caspase-4 activity of PBMCs of N and H patients can be enhanced by very high concentrations. Regarding the much lower concentration of indoxyl sulfate that was found in the plasma of HD patients (up to 300 µM), it is very unlikely that indoxyl sulfate is the cause of increased caspase activity in H patients [33]. Of course, it cannot be excluded that caspase-4 activity is also triggered by other factors that have not been characterized yet.

Therefore, our study broadens the knowledge of inflammatory routes in hypervolemia. Without a doubt, hypervolemia contributes to inflammation in CKD5D-patients. Of course, we are still not sure which are the main factors triggering inflammation, but we can be sure that hypervolemic inflammation is not only linked to enhanced induction but also to disturbed resolution of inflammation. Exact molecular analysis of the RelB action in hypervolemia is required to support our findings. It is possible that in the future, caspase-4 activity can be established as a reliable marker of the intracellular load of endotoxin in HD patients.

### Limitations of the Study

The main drawback of this study is the small sample size. It was designed as a cross-sectional study to gain insight into the inflammation-driven mechanism that dominates signaling in hypervolemic HD patients. We further appreciate that because of the multimorbid nature of CKD5-D patients, signaling pathways may be influenced by different factors that may affect assays and results. In this regard, we found indications of a lack of anti-inflammatory signaling via RelB and elevated caspase-4 activity, which may be representative of elevated intracellular endotoxin loads in such patients. Although the caspase-4/-5 assay used in this study was validated in an earlier study [33], we must admit that this assay—because of the very broad substrate spectrum of different caspases—may also detect to caspase-8 activity to some extent. Nevertheless, the caspase activity measured in this study appears to be predominantly caspase-4-dependent.

## 4. Materials and Methods

### 4.1. Study Population

To analyze hypervolemic effects in HD, we initiated a cross-sectional study, originally enrolling 46 patients on maintenance hemodialysis at the Nephrology outpatient dialysis center of the Department of Internal Medicine II of the University of Halle-Wittenberg, Germany. Inclusion criteria included age >18 years and a history of hemodialysis treatment >12 weeks. Patients with active malignancy, active infections and neurological disorders preventing them from providing consent were excluded from the study. Patients who failed to reach a minimum ultrafiltration volume of 2 liters were also excluded from the study. According to these criteria, 1 patient had to be excluded because of an acute infection, 3 patients did not reach the minimum ultrafiltration volume, 1 patient changed to another dialysis center and 1 patient withdrew his consent. Thus, among 46 patients, 40 could be enrolled in the study. A total of 11 (26%) patients were dialyzed through permanent catheters, 26 (62%) patients were dialyzed through arteriovenous fistulas and 5 (12%) patients underwent an arteriovenous graft. The use of high-flux hemodialysis (polysulfone membrane filters) 3 times per week for 4–5 h per session is the standard of care in our dialysis center. Only one patient was dialyzed with a low-flux dialyzer because he had an allergic reaction to a polysulfone membrane in advance of the study. Reasons for kidney failure included glomerulonephritis (17.5%), diabetic nephropathy (25.0%), interstitial nephritis (2.5%) and ischemic nephropathy (17.5%), among others (37.5%). Fluid status and bioelectrical impedance vector analyses [25] were conducted by Nutriguard-MS (Data Input GmbH, Pöcking, Germany). Normohydration was defined as the range of impedance vectors falling within the reference 75% tolerance interval, and overhydration (hypervolemia) was defined by a Piccoli vector diagram >75th percentile. The fluid status was assessed during all hemodialysis sessions based on clinical symptoms and dialysis device parameters. For this study, an additional measurement of the fluid status was performed by bioelectrical impedance analysis after the last week of dialysis sessions, i.e., before the patient proceeded to the long interdialytic interval at home. Immunobiologic samples were taken after the long intradialytic interval and before the start of first week of dialysis. Heparin was used as an anticoagulant drug during hemodialysis treatment in all patients. CRP, albumin creatinine and hemoglobin were measured monthly in a certified clinical laboratory using standardized methods. The mean residual diuresis was 449 ± 652 mL/d, but 19 dialysis patients (47.5%) did not demonstrate any urine production. The mean UF volume was 2527 ± 712 mL per session. Blood pressure was measured once before the start of dialysis, during dialysis sessions (2–3 times) and at the end of the dialysis sessions using the automatic blood pressure device of the hemodialysis system. Nine dialysis patients (22.5%) had one hypotensive episode during the dialysis study. The prevalence of peripheral artery disease (PAD) was 12.5% (5 cases). PAD did not differ between the two groups. This immunosuppressive regimen did not differ between the two groups (N: 5 of 23; H: 3 of 17 patients; *p* = 0.999).

This study was conducted according to the Declaration of Helsinki. Written informed consent was obtained from all study subjects, and the study protocol was approved by the local ethics committee.

### 4.2. PBMC Isolation

PBMCs were isolated from blood samples anticoagulated with EDTA by Ficoll density gradient centrifugation (GE Healthcare, Chicago, IL, USA). The quality of isolated cells was tested by 7-AAD staining. The vitality of PBMCs was 99.2% ± 1.6 for N patients and 99.1% ± 0.5 for H patients.

### 4.3. RNA/cDNA/qPCR

For the detection of caspase-4 and cytokine transcripts, RNA was isolated from PBMC lysates using a Quick RNA MiniPrep Isolation Plus Kit (ZymoResearch, Freiburg, Germany). The RNA concentration and quality (260/280 ratio: 1.8 ± 0.1) was tested using the Nanodrop technique (PEQLAB Biotechnologie GmbH, Erlangen, Germany). Equal amounts of RNA (50 ng) were reverse-transcribed using a High Capacity cDNA Reverse Transcription Kit (Thermo Fisher Scientific, Darmstadt, Germany).

TNF-α (Hs01113624_m1), TNFRSF1a (Hs01042313_m1), TNFRSF1b (Hs00961749_m1), IL-6 (Hs00985639_m1) and RPL37A (Hs01102345_m1) mRNA expressions were analyzed using TaqMan probes (Thermo Fisher Scientific) in combination with qPCRBIO Probe Mix High-ROX (Nippon Genetic Europe, Düren, Germany). The samples were processed in duplicate on a StepOnePlus Cycler (Thermo Fisher Scientific). Data were normalized by RPL37A and related to healthy control donor RNA. Thus, results are expressed as x-fold difference compared to the healthy control.

### 4.4. Analysis of TNF-α Secretion by Flow Cytometry

A total of 0.5 × 10^6^ PBMCs were incubated under basal and stimulated conditions (LPS (0127:B8, 10 µg/mL (Sigma-Aldrich, Steinheim, Germany))) in the presence of monensin (end concentration: 0.5 fold; Biolegend, Amsterdam, The Netherlands). After 14 h, cells were stained with viability dye (405/450, Miltenyi Biotec, Bergisch-Gladbach, Germany), followed by surface staining of cells with anti-CD14 and anti-CD3 (both from Miltenyi Biotec). The cells were fixed using 1% paraformaldehyde solution. After saponin treatment, PBMCs were stained with anti-TNF-α (Miltenyi Biotec) or a corresponding isotype control (Biolegend). Samples were analyzed on a MACS Quant analyzer (Miltenyi Biotec) using MACS Quantify software2.5.

### 4.5. Analysis of RelA and RelB by Phospho-Flow Cytometry

A total of 0.5 × 10^6^ PBMCs were incubated under basal and stimulated conditions (LPS (0127:B8, 10 µg/mL (Sigma-Aldrich)). After 30 min, the cells were fixed using 4% paraformaldehyde solution, followed by permeabilization of cells using ice-cold PhosFlow Perm Buffer III (BD Biosciences, Heidelberg, Germany). Afterwards, cells were stained with anti-CD86 and anti-CD4 (both from Thermo Fisher Scientific), anti-phospho-NF-kB (RelA, pS529) (BD Biosciences) and anti-phospho-RelB (pS552) (Thermo Fisher Scientific). Samples were analyzed using a MACS Quant analyzer (Miltenyi Biotec) with MACS Quantify software. Gates were set according to FMO controls.

### 4.6. Caspase-4 Assay

A Caspase-Glo^®^1 Inflammasome Assay was purchased from Promega (Heidelberg, Germany). A total of 0.5 × 10^6^ PBMCs were seeded in quadruplicate in a white, opaque-walled 96-well plate (Greiner, Frickenhausen, Germany). Cells were incubated for 20 h with or without the caspase-4 inhibitor (Ac-LEVD-CHO, 2 µM) and the caspase-8 inhibitor (Ac-IETD-CHO, 2 µM) (both from Merck Millipore, Darmstadt, Germany), respectively. After the addition of the Caspase Glo Reagent (the peptide substrate to detect inflammatory caspases is Z-WEHD), the plate was incubated for 60 min, and luminance was measured using an Infinite M200 Pro analyzer (Tecan, Crailsheim, Germany).

### 4.7. Endotoxin Detection

Endotoxin was photometrically detected using a Pierce™ Chromogenic Endotoxin Quant kit (Thermo Fisher Scientific). Data were analyzed on an ELX808 microplate reader (Bio-Tek Inc., Berlin, Germany).

### 4.8. Cytokine Analysis

IL-6 and TNF-α serum levels were analyzed using high-sensitivity ELISAs, whereas TNF-α in cell culture supernatants was measured using common TNF-α kits (all Tecan).

### 4.9. Statistics

Results are expressed as mean ± SD. All continuous variables were controlled for normal distribution using the D’Agostino–Pearson omnibus test. Continuous data were compared by Mann–Whitney test or by one-way ANOVA followed by Fisher’s LSD post hoc test as appropriate. All calculations were carried out using SPSS 21.0 (SPSS Inc., Chicago, IL, USA) or GraphPad Prism 9.2.0 statistical software (GraphPad Software Inc., La Jolla, CA, USA). The level of significance was set at *p* < 0.05.

## Figures and Tables

**Table 1:** Demographic data of normo- (N) and hypervolemic patients (H).

	N (n = 23)	H (n = 17)	Statistics
Age (years)	57.6 ± 14.3	67.5 ± 10.2	0.020
Sex (f, %)	60.9	52.9	0.749
Diabetes (%)	17.4	52.9	0.038
BMI (kg/m^2^)	26.7 ± 8.1	29.4 ± 6.9	0.184
Dialysis vintage (years)	7.6 ± 5.7	7.1 ± 10.9	0.073
Kt/V	1.6 ± 0.4	1.2 ± 0.3	0.002
CRP (mg/dL)	5.2 ± 6.3	14.2 ± 16.1	0.020
Albumin (g/L)	40.1 ± 3.2	38.6 ± 4.7	0.256
Hemoglobin (mmol/L)	6.9 ± 0.7	7.0 ± 0.9	0.948
Creatinine (µmol/L)	930 ± 210	679 ± 246	0.001
ECW (L)	14.9 ± 6.2	20.4 ± 4.9	0.001
ICW (L)	22.5 ± 3.8	24.8 ± 4.6	0.084
TBW (L)	37.3 ± 9.4	45.2 ± 9.2	0.009
Interdialytic weight gain (kg)	1.9 ± 2.0	3.0 ± 1.2	0.062
Hypertensive drugs (n)	2.4 ± 1.3	2.4 ± 1.1	0.836
Diuretic drugs (n)	0.7 ± 0.9	1.2 ± 1.0	0.126
Systolic BP (mm Hg)	127 ± 27	143 ± 31	0.093
Diastolic BP (mm Hg)	69 ± 13	70 ± 20	0.806

ECW: extracellular water; ICW: intracellular water; TBW: total body water; CRP: C-reactive protein.

**Table 2:** Leucocyte subtypes according to hydration status.

	N (n = 23)	H (n = 17)	Statistics
CD45+ (×10^6^/mL)	9.1 ± 3.0	10.8 ± 4.3	0.079
Granulocytes (×10^6^/mL)	5.7 ± 1.9	7.3 ± 3.2	0.065
CD14+ Monocytes (×10^6^/mL)	0.7 ± 0.4	0.8 ± 0.3	0.381
CD3+ Lymphocytes (×10^6^/mL)	1.3 ± 0.6	1.2 ± 0.5	0.728
CD4+ (×10^6^/mL)	0.7 ± 0.4	0.6 ± 0.3	0.636
CD8+ (×10^6^/mL)	0.4 ± 0.2	0.5 ± 0.3	0.216
Treg+ (CD25+CD127low, ×10^6^/mL)	0.02 ± 0.02	0.02 ± 0.02	0.120

**Table 3:** Frequency of CD4-positive cells staining positive for RelA (pS529) and RelB (pS552).

	%CD4+RelA (pS529)+ (Basal)	%CD4+RelA (pS529)+ (LPS)	%CD4+RelB (pS552)+ (Basal)	%CD4+RelB (pS552)+ (LPS)
N	10.8 ± 10.4	16.2 ± 13.4	57.0 ± 24.2	78.9 ± 19.7
H	16.1 ± 13.3	18.8 ± 15.5	61.2 ± 28.5	61.5 ± 28.0
*p*-value	0.112	0.568	0.656	0.017

## Data Availability

Data is contained within the article.

## References

[1] Khan Y.H., Sarriff A., Adnan A.S., Khan A.H., Mallhi T.H. (2016). Chronic Kidney Disease, Fluid Overload and Diuretics: A Complicated Triangle. PLoS ONE.

[2] Hung S.-C., Kuo K.-L., Peng C.-H., Wu C.H., Lien Y.C., Wang Y.C., Tarng D.C. (2014). Volume overload correlates with cardiovascular risk factors in patients with chronic kidney disease. Kidney Int..

[3] Vega A., Abad S., Macías N., Aragoncillo I., García-Prieto A., Linares T., Luño J. (2018). Any grade of relative overhydration is associated with long-term mortality in patients with Stages 4 and 5 non-dialysis chronic kidney disease. Clin. Kidney J..

[4] Dekker M.J.E., Marcelli D., Canaud B.J., Carioni P., Wang Y., Grassmann A. (2017). Impact of fluid status and inflammation and their interaction on survival: A study in an international hemodialysis patient cohort. Kidney Int..

[5] Damman K., van Deursen V.M., Navis G., Voors A.A., van Veldhuisen D.J., Hillege H.L. (2009). Increased central venous pressure is associated with impaired renal function and mortality in a broad spectrum of patients with cardiovascular disease. J. Am. Coll. Cardiol..

[6] Wang Y., Gao L. (2022). Inflammation and Cardiovascular Disease Associated With Hemodialysis for End-Stage Renal Disease. Front. Pharmacol..

[7] Vaziri N.D. (2012). CKD impairs barrier function and alters microbial flora of the intestine: A major link to inflammation and uremic toxicity. Curr. Opin. Nephrol. Hypertens..

[8] McIntyre C.W., Harrison L.E.A., Eldehni M.T., Jefferies H.J., Szeto C.C., John S.G., Li P.K. (2011). Circulating endotoxemia: A novel factor in systemic inflammation and cardiovascular disease in chronic kidney disease. Clin. J. Am. Soc. Nephrol..

[9] Terpstra M.L., Singh R., Geerlings S.E., Bemelman F.J. (2016). Measurement of the intestinal permeability in chronic kidney disease. World J. Nephrol..

[10] Beberashvili I., Azar A., Sinuani I., Shapiro G., Feldman L., Stav K., Averbukh Z. (2014). Bioimpedance phase angle predicts muscle function, quality of life and clinical outcome in maintenance hemodialysis patients. Eur. J. Clin. Nutr..

[11] Zelová H., Hošek J. (2013). TNF-α signalling and inflammation: Interactions between old acquaintances. Inflamm. Res..

[12] Kapadia S.R., Oral H., Lee J., Nakano M., Taffet G.E., Mann D.L. (1997). Hemodynamic regulation of tumor necrosis factor-alpha gene and protein expression in adult feline myocardium. Circ. Res..

[13] Bradley J.R. (2008). TNF-mediated inflammatory disease. J. Pathol..

[14] Wang D., Baldwin A.S. (1998). Activation of nuclear factor-kappaB-dependent transcription by tumor necrosis factor-alpha is mediated through phosphorylation of RelA/p65 on serine 529. J. Biol. Chem..

[15] Wang D., Westerheide S.D., Hanson J.L., Baldwin A.S. (2000). Tumor necrosis factor alpha-induced phosphorylation of RelA/p65 on Ser529 is controlled by casein kinase II. J. Biol. Chem..

[16] Senftleben U., Cao Y., Xiao G., Greten F.R., Krahn G., Bonizzi G., Karin M. (2001). Activation by IKKalpha of a second, evolutionary conserved, NF-kappa B signaling pathway. Science.

[17] Marienfeld R., Berberich-Siebelt F., Berberich I., Denk A., Serfling E., Neumann M. (2001). Signal-specific and phosphorylation-dependent RelB degradation: A potential mechanism of NF-kappaB control. Oncogene.

[18] Ulrich C., Wilke A., Schleicher N., Girndt M., Fiedler R. (2020). Hypervolemia-Induced Immune Disturbances Do Not Involve IL-1ß but IL-6 and IL-10 Activation in Haemodialysis Patients. Toxins.

[19] Zamyatina A., Heine H. (2020). Lipopolysaccharide Recognition in the Crossroads of TLR4 and Caspase-4/11 Mediated Inflammatory Pathways. Front. Immunol..

[20] Piccoli A. (2014). Estimation of fluid volumes in hemodialysis patients: Comparing bioimpedance with isotopic and dilution methods. Kidney Int..

[21] Piccoli A., Codognotto M., Piasentin P., Naso A. (2014). Combined evaluation of nutrition and hydration in dialysis patients with bioelectrical impedance vector analysis (BIVA). Clin. Nutr..

[22] Tsai Y.-C., Tsai J.-C., Chen S.-C., Chiu Y.W., Hwang S.J., Hung C.C., Chen H.C. (2014). Association of fluid overload with kidney disease progression in advanced CKD: A prospective cohort study. Am. J. Kidney Dis..

[23] Levin N.W., Kotanko P., Eckardt K.-U., Kasiske B.L., Chazot C., Cheung A.K., London G.M. (2010). Blood pressure in chronic kidney disease stage 5D-report from a Kidney Disease: Improving Global Outcomes controversies conference. Kidney Int..

[24] Canaud B., Stephens M.P., Nikam M., Etter M., Collins A. (2021). Multitargeted interventions to reduce dialysis-induced systemic stress. Clin. Kidney J..

[25] Zsom L., Faludi M., Fülöp T., Dossabhoy N.R., Rosivall L., Tapolyai M.B. (2019). The association of overhydration with chronic inflammation in chronic maintenance hemodiafiltration patients. Hemodial. Int..

[26] Jang D.-I., Lee A.-H., Shin H.-Y., Song H.R., Park J.H., Kang T.B., Yang S.H. (2021). The Role of Tumor Necrosis Factor Alpha (TNF-α) in Autoimmune Disease and Current TNF-α Inhibitors in Therapeutics. Int. J. Mol. Sci..

[27] Parameswaran N., Patial S. (2010). Tumor necrosis factor-α signaling in macrophages. Crit. Rev. Eukaryot. Gene Expr..

[28] Caldwell A.B., Cheng Z., Vargas J.D., Birnbaum H.A., Hoffmann A. (2014). Network dynamics determine the autocrine and paracrine signaling functions of TNF. Genes Dev..

[29] Baud V., Collares D. (2016). Post-Translational Modifications of RelB NF-κB Subunit and Associated Functions. Cells.

[30] Saccani S., Pantano S., Natoli G. (2003). Modulation of NF-kappaB activity by exchange of dimers. Mol. Cell.

[31] McMillan D.H., Woeller C.F., Thatcher T.H., Spinelli S.L., Maggirwar S.B., Sime P.J., Phipps R.P. (2013). Attenuation of inflammatory mediator production by the NF-κB member RelB is mediated by microRNA-146a in lung fibroblasts. Am. J. Physiol. Lung Cell Mol. Physiol..

[32] Ulrich C., Kneser L., Fiedler R., Beckert J., Wildgrube S., Seibert E., Girndt M. (2021). Pyroptosis: A Common Feature of Immune Cells of Haemodialysis Patients. Toxins.

[33] Kim H.Y., Yoo T.-H., Hwang Y., Lee G.H., Kim B., Jang J., Lee W.W. (2017). Indoxyl sulfate (IS)-mediated immune dysfunction provokes endothelial damage in patients with end-stage renal disease (ESRD). Sci. Rep..

